# Integrated analysis of single‐cell RNA‐seq dataset and bulk RNA‐seq dataset constructs a prognostic model for predicting survival in human glioblastoma

**DOI:** 10.1002/brb3.2575

**Published:** 2022-04-16

**Authors:** Wenwen Lai, Defu Li, Jie Kuang, Libin Deng, Quqin Lu

**Affiliations:** ^1^ Jiangxi Provincial Key Laboratory of Preventive Medicine Nanchang University Nanchang China; ^2^ Department of Biostatistics and Epidemiology School of Public Health Nanchang University Nanchang China

**Keywords:** glioblastoma, overall survival, prognostic model, single‐cell RNA‐seq, bulk RNA‐seq

## Abstract

**Background:**

Glioblastoma (GBM) is the most common primary malignant brain tumor in adults. For patients with GBM, the median overall survival (OS) is 14.6 months and the 5‐year survival rate is 7.2%. It is imperative to develop a reliable model to predict the survival probability in new GBM patients. To date, most prognostic models for predicting survival in GBM were constructed based on bulk RNA‐seq dataset, which failed to accurately reflect the difference between tumor cores and peripheral regions, and thus show low predictive capability. An effective prognostic model is desperately needed in clinical practice.

**Methods:**

We studied single‐cell RNA‐seq dataset and The Cancer Genome Atlas‐glioblastoma multiforme (TCGA‐GBM) dataset to identify differentially expressed genes (DEGs) that impact the OS of GBM patients. We then applied the least absolute shrinkage and selection operator (LASSO) Cox penalized regression analysis to determine the optimal genes to be included in our risk score prognostic model. Then, we used another dataset to test the accuracy of our risk score prognostic model.

**Results:**

We identified 2128 DEGs from the single‐cell RNA‐seq dataset and 6461 DEGs from the bulk RNA‐seq dataset. In addition, 896 DEGs associated with the OS of GBM patients were obtained. Five of these genes (*LITAF*, *MTHFD2*, *NRXN3*, *OSMR*, and *RUFY2*) were selected to generate a risk score prognostic model. Using training and validation datasets, we found that patients in the low‐risk group showed better OS than those in the high‐risk group. We validated our risk score model with the training and validating datasets and demonstrated that it can effectively predict the OS of GBM patients.

**Conclusion:**

We constructed a novel prognostic model to predict survival in GBM patients by integrating a scRNA‐seq dataset and a bulk RNA‐seq dataset. Our findings may advance the development of new therapeutic targets and improve clinical outcomes for GBM patients.

## Graphical abstract

We constructed a novel prognostic model to predict survival in glioblastoma patients by integrating single‐cell RNA‐seq dataset and bulk RNA‐seq dataset. These results may advance the development of new therapeutic targets and improve clinical outcomes for glioblastoma patients.

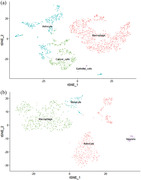

## 1 INTRODUCTION

Glioblastoma (GBM) is the most common primary malignant brain tumor in adults, accounting for 48.6% of malignant tumors in the central nervous system and 14.5% of all tumors (Ostrom et al., 2020). The median overall survival (OS) time is around 14.6 months for patients diagnosed with GBM, with only a 5‐year survival rate of 7.2% (Lynes et al., 2020; Ostrom et al., 2020). Currently, the main treatment measures for GBM include radiotherapy, chemotherapy, and surgical resection (Fabian et al., 2019). Unfortunately, little progress has been made toward prolonging survival in GBM despite considerable effort in improving treatments over the past decades (Alexander & Cloughesy, 2017). The OS of each GBM patient is a crucial factor in developing a personalized treatment plan. Therefore, it is imperative to develop a reliable tool to predict the survival probability for patients with newly diagnosed GBM.

With the advancement of high‐throughput technologies, RNA sequencing (RNA‐seq) from bulk tissue has become indispensable for transcriptome‐wide analysis (Stark et al., 2019). Many public databases have been established, including The Cancer Genome Atlas (TCGA, https://cancergenome.nih.gov/) and Gene Expression Omnibus (GEO, https://www.ncbi.nlm.nih.gov/geo/). These databases enable scientists to investigate the relationship between the prognosis of diseases and gene expression profiles. Studies are in mounting numbers being reported to identify biomarkers associated with prognosis for GBM patients (Wang et al., 2019; Zhao et al., 2021; Zhou et al., 2021). However, the expression of genes obtained from bulk tissue does not reflect their expression in individual cells, which leads to the high heterogeneity of GBM being masked.

The development of single‐cell RNA sequencing (scRNA‐seq) in recent years has significantly expanded our knowledge about biological systems. As an emerging technology, scRNA‐seq has been applied increasingly to explore extensive intratumoral heterogeneity (Kinker et al., 2020; Patel et al., 2014; Peng et al., 2019). Compared to calculating the average gene expression in all the cells, scRNA‐seq allows the evaluation of gene expression at a single‐cell resolution, which greatly compensates for the shortage of RNA‐seq from bulk tissue (G. Chen et al., 2019). In addition, scRNA‐seq analysis enables researchers to discover critical genes that are characteristic of cancer cells (Kulkarni et al., 2019). In this study, we studied a scRNA‐seq dataset and a bulk RNA‐seq dataset and integrated them to construct a novel prognostic model for predicting survival in GBM.

## 2 MATERIALS AND METHODS

### 2.1 Acquisition of bulk RNA‐seq dataset and scRNA‐seq dataset in GBM patients

We included the scRNA‐seq dataset and three bulk RNA‐seq datasets of human GBM samples in our study. We first obtained the gene expression dataset and related clinical information of GBM patients from The Cancer Genome Atlas‐glioblastoma multiforme (TCGA‐GBM) dataset. The gene expression dataset and the clinical information from GSE43378 were collected from the GEO database. In addition, the RNA sequencing dataset and corresponding clinical information that contained 693 samples (dataset ID: mRNAseq_693) were downloaded from Chinese Glioma Genome Atlas (CGGA; http://www.cgga.org.cn) database. We first performed data clean‐up. We excluded cases that do not have follow‐up time or survival status, as well as the ones that had clinical information but no corresponding RNA‐seq data. According to the exclusion criteria, a total of 152 tumor samples and five normal controls in the TCGA‐GBM dataset were enrolled in the study and selected as the training dataset, and a total of 50 tumor samples in the GSE43378 dataset and a total of 133 GBM samples in the mRNAseq_693 dataset were enrolled and selected as the validation datasets. The scRNA‐seq dataset with a total of 3589 cells from four human primary GBM samples from the GSE84465 dataset was acquired from the GEO database. Among the 3589 cells, 2343 were from tumor cores and 1246 were from peripheral regions, with a reading depth of 10× genomics based on Illumina NextSeq 500.

### 2.2 The processing of GBM scRNA‐seq dataset

We analyzed 2343 cells from tumor cores as follows. We used the Seurat package in R 4.0.0 to perform quality control, statistical analysis, and explore the scRNA‐seq dataset (Gribov et al., 2010). We calculated the percentage of mitochondrial genes with the PercentageFeatureSet function and elucidated the relationship between the sequencing depth, the mitochondrial gene sequences, and total intracellular sequences through correlation analysis. We cleaned up data according to the following quality control criteria: first, genes detected in < 3 cells were omitted; second, cells with < 100 total detected genes were excluded; third, cells with ≥ 5% mitochondria‐expressed genes were discarded; and last, cells with nuclei gene counts < 200 or > 6000 were excluded. We normalized the gene expression of the remaining cells with the LogNormalize method, and we identified the top 1500 genes with highly variable features by variance analysis. We then performed principal component analysis (PCA) to identify significantly available dimensions with a *p*‐value < .05 based on the expression of these genes (Ringnér, 2008). We next applied the *t*‐distributed stochastic neighbor embedding (tSNE) algorithm to reduce dimensionality with 20 initial PCs and perform cluster classification analysis (Kobak & Berens, 2019). With the criteria of log_2_ [fold change (FC)] > 0.25 and an adjusted *p*‐value < .05, marker genes in each cluster were obtained. Clusters were annotated through the “SingleR” package based on these marker genes (Aran et al., 2019).

Then, 1246 cells from peripheral regions were analyzed as described before, except that the cells with nuclei gene counts < 200 or > 4000 were excluded rather than cells with sequencing number < 200 or nuclei gene counts > 6000.

### 2.3 The identification of DEGs from the scRNA‐seq dataset and TCGA‐GBM dataset

In the GBM scRNA‐seq dataset, cancer cells were selected as representative tumor cores after annotation, and neurons were selected as representative peripheral regions. Then, the differentially expressed genes (DEGs) between cancer cells and neurons were identified by the “DEsingle” package (Miao et al., 2018). Genes with |log_2_ FC| > 2 and an adjusted *p*‐value < .05 were considered DEGs. For the TCGA‐GBM dataset, first, log_2_ transformation was employed to generate expression profiles, and then the genes between tumor samples and normal controls were used for differentially expressed analysis using the “Limma” package (Ritchie et al., 2015). Genes with |log_2_ FC| > 1 and *p‐*value < .05 were defined as DEGs.

### 2.4 Enrichment analysis of Gene Ontology functions and Kyoto Encyclopedia of Genes and Genomes pathways for DEGs

To investigate the biological implications of DEGs identified from both the GBM scRNA‐seq and TCGA‐GBM datasets, we performed an intersection of the datasets. We then conducted Gene Ontology (GO) function and Kyoto Encyclopedia of Genes and Genomes (KEGG) pathway analysis using WebGestalt (WebGestalt: WEB‐based GEne SeT AnaLysis Toolkit, RRID:SCR_006786). A gene set at *p* < .05 and false discovery rate (FDR) < 0.05 was considered to be significantly enriched.

### 2.5 Analysis of DEGs associated with overall survival in GBM patients

First, univariate Cox proportional hazards regression analysis was used to assess the relationship between the expression of genes and the OS of patients in the TCGA‐GBM dataset. Genes with hazard ratio (HR)≠1 and *p* < .05 were defined as genes associated with OS. Then, DEGs associated with OS were obtained by overlapping genes associated with OS and DEGs from both the GBM scRNA‐seq and TCGA‐GBM datasets.

### 2.6 Prognostic model construction

The DEGs associated with OS were regarded as candidate genes for constructing a prognostic model. Then, we conducted least absolute shrinkage and selection operator (LASSO) Cox penalized regression analysis using the R package “glmnet” (Friedman et al., 2010), and the genes with nonzero coefficients were selected to establish a risk score prognostic model. Based on the results of LASSO Cox penalized regression analysis, we calculated the risk score for each GBM patient in the training dataset.

### 2.7 Prognostic model validation

After constructing the risk score prognostic model, we used one independent dataset GSE43378 including 50 patients with complete OS information from GEO and the other independent dataset mRNAseq_693 including 133 patients with complete OS information from CGGA to validate the model, respectively. First, we performed time‐dependent receiver operating characteristic (ROC) curve analysis to predict the 12‐, 15‐, and 18‐month survival using the R package “survivalROC” (Lorent et al., 2014). Then, based on the median risk score, we divided GBM patients into high‐ and low‐risk groups. We performed Kaplan–Meier survival analysis to determine the association between the risk score prognostic model and the OS of GBM patients. The significance of differences in survival between the two groups was determined by the log‐rank test.

## 3 RESULTS

### 3.1 Identification of cancer cells and neurons in the GBM scRNA‐seq dataset

**FIGURE 1:**
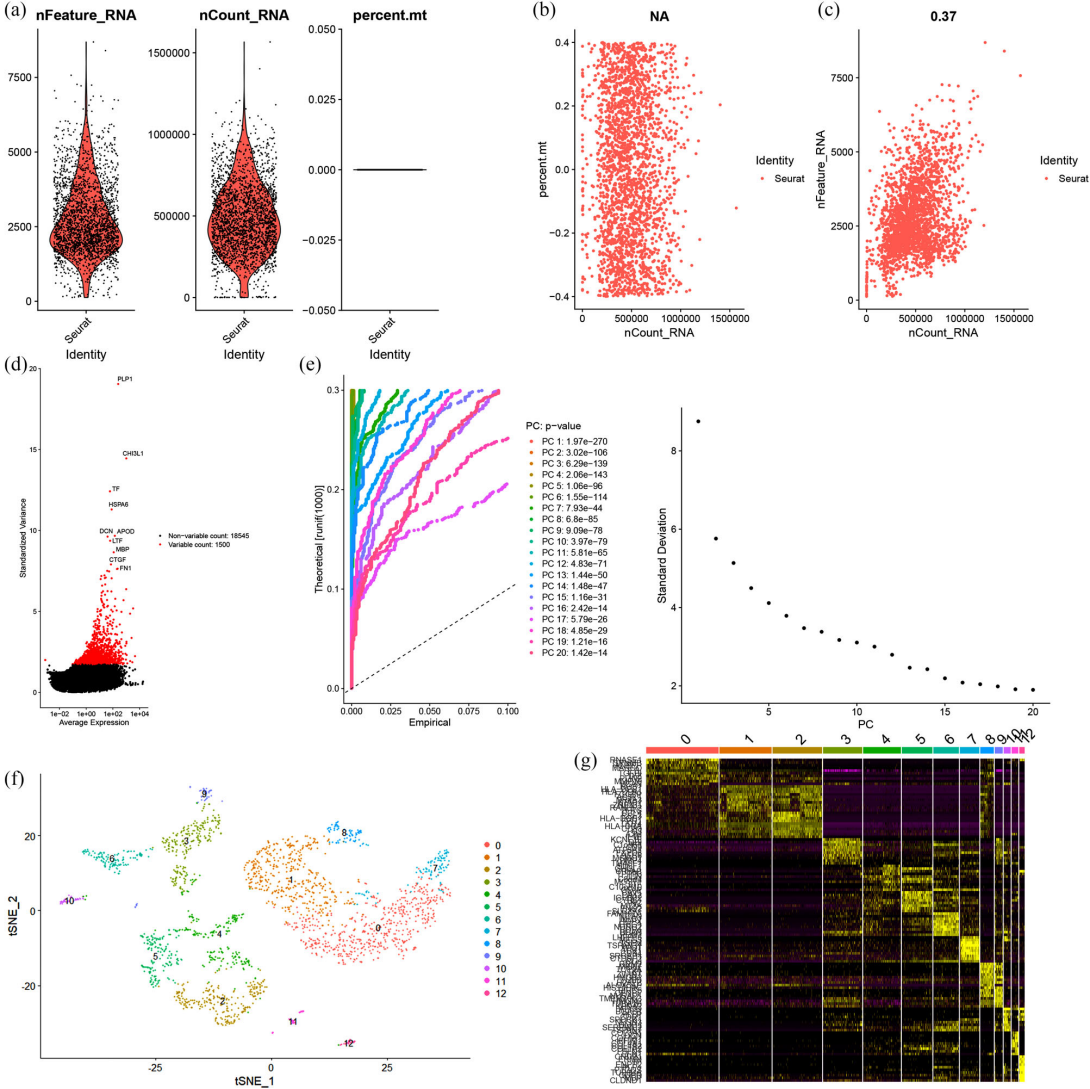
The processing of cells from tumor cores in the glioblastoma (GBM) scRNA‐seq dataset. (a) Thirty‐eight nonconforming cells were filtered out during quality control and normalization, and 2305 cells were screened for further analysis. (b) Correlation analysis of sequencing depth and mitochondrial gene sequences. (c) Correlation analysis of sequencing depth and total intracellular sequences. (d) Among the 18,545 genes analyzed, 17,045 showed low and 1500 showed high intercellular variation. (e) Twenty principal components (PCs) with significant differences were identified with *p* < .05. (f) Two thousand three hundred five cells were divided into 13 separate clusters. (g) Heatmap displaying the top 10 marker genes in each cluster

Thirty‐eight nonconforming cells were excluded, and 2305 cells were preserved for further analysis after quality control from tumor cores (Figure 1a). We performed correlation analysis and found that there appeared to be no correlation between sequencing depth and mitochondrial gene sequences (Figure 1b). However, there was a significant positive correlation between the sequencing depth and total intracellular sequences (*r* = 0.37, Figure 1c). We also found that among the total of 18,545 genes analyzed, 1500 had high variation and 17,045 had low intercellular variation (Figure 1d). For PCA analysis, we picked 20 principal components (PCs) that have a *p*‐value < .05 for subsequent analysis (Figure 1e). Then, we applied the tSNE algorithm and successfully classified the cells from tumor cores into 13 separate clusters (Figure 1f). We identified a total of 13,616 marker genes from all 13 clusters, and the top 10 marker genes from each cluster were presented in the heatmap (Figure 1g). We annotate clusters with singleR based on the expression of these marker genes (Figure 3a). We determined that the Clusters 0, 1, 7, and 8, containing 1176 cells, were macrophages; Clusters 2 and 4, containing 450 cells, were GBM cancer cells; Clusters 3, 5, 6, 9, 10, and 12, containing 637 cells, were astrocytes; and Cluster 11, containing 42 cells, was endothelial cells.

**FIGURE 2:**
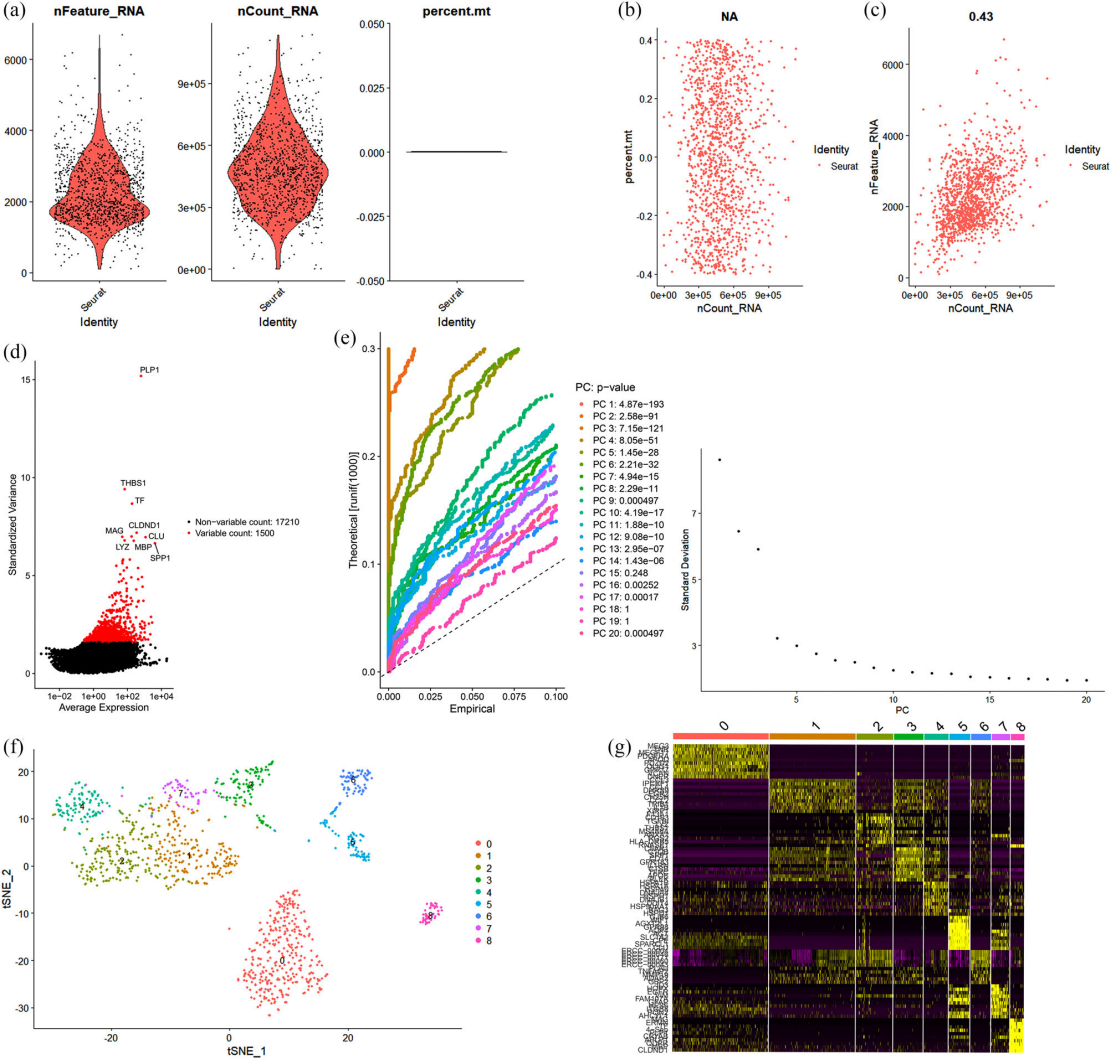
The processing of cells from peripheral regions in the glioblastoma (GBM) scRNA‐seq dataset. (a) Fifty‐three nonconforming cells were filtered out during quality control and normalization, and 1193 cells were screened for further analysis. (b) Correlation analysis of sequencing depth and mitochondrial gene sequences. (c) Correlation analysis of sequencing depth and total intracellular sequences. (d) Among the total of 17210 genes analyzed, 15710 showed low intercellular variation, while 1500 showed high variation. (e) Seventeen principal components (PCs) with significant differences were identified with *p* < .05. (f) A total of 1193 cells were divided into nine separate clusters. (g) Heatmap displaying the top 10 marker genes in each cluster

**FIGURE 3:**
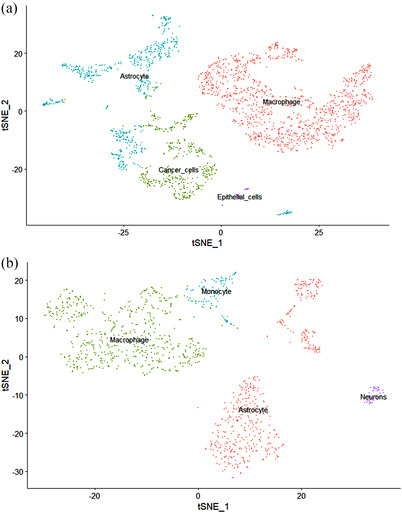
Cell annotation by singleR corresponding to the composition of the marker genes in each cluster. (a) All 13 clusters of cells from tumor cores in glioblastoma (GBM) scRNA‐seq dataset were annotated. (b) All nine clusters of cells from peripheral regions in GBM scRNA‐seq dataset were annotated

A total of 1193 cells were screened, and 53 nonconforming cells were excluded for further analysis after quality control from peripheral regions (Figure 2a). We found that though the sequencing depth did not have correlation with mitochondrial gene sequences (Figure 2b), it showed a significant positive correlation with total intracellular sequences (*R* = 0.43, Figure 2c). Among the 17,210 genes analyzed, 500 had high variation and 15,710 had low intercellular variation (Figure 2d). We executed PCA and selected 17 PCs with a *p*‐value < .05 for subsequent analysis (Figure 2e). Then, we performed the tSNE algorithm and divided the cells from peripheral regions into nine separate clusters (Figure 2f). We have identified a total of 6748 marker genes from all nine clusters, and the top 10 marker genes from each cluster were laid out in the heatmap (Figure 2g). All clusters were annotated by singleR based on the expression of these marker genes (Figure 3b). Clusters 0, 5, and 6, containing 482 cells, were annotated as astrocytes; Clusters 1, 2, 4, and 7, containing 559 cells, were classified as macrophages; Cluster 3, containing 105 cells, was annotated as monocytes; and Cluster 8, containing 47 cells, was classified as neurons.

### 3.2 DEGs from scRNA‐seq dataset and TCGA‐GBM dataset

A total of 450 cancer cells were selected as representative tumor cores, and 47 neurons were selected as representative peripheral regions in the GBM scRNA‐seq dataset. We obtained 2128 DEGs at |log_2_ FC| > 2 and an adjusted *p*‐value < .05 between these cancer cells and neurons. In addition, 6461 DEGs at |log2 FC| > 1 and *p* < .05 were identified between 152 tumor samples and five normal controls in the TCGA‐GBM dataset.

### 3.3 Enrichment analysis of GO functions and KEGG pathways

There were 896 genes in the intersection of the identified DEGs from the GBM scRNA‐seq and TCGA‐GBM datasets. The KEGG analysis on the 896 genes suggested that they were enriched in signaling pathways such as the MAPK signaling pathway, the apelin signaling pathway, and pathways involved in circadian entrainment (Figure S1).

### 3.4 DEGs associated with OS in GBM patients

We preliminarily identified 1418 genes that are linked to the OS of patients using univariate Cox proportional hazards regression analysis on the TCGA‐GBM dataset. We found that 43 genes were at the intersection of genes associated with OS and DEGs from the GBM scRNA‐seq dataset and TCGA‐GBM dataset. These genes were chosen as candidate genes for constructing a prognostic model.

### 3.5 Construction of the prognostic model

**FIGURE 4:**
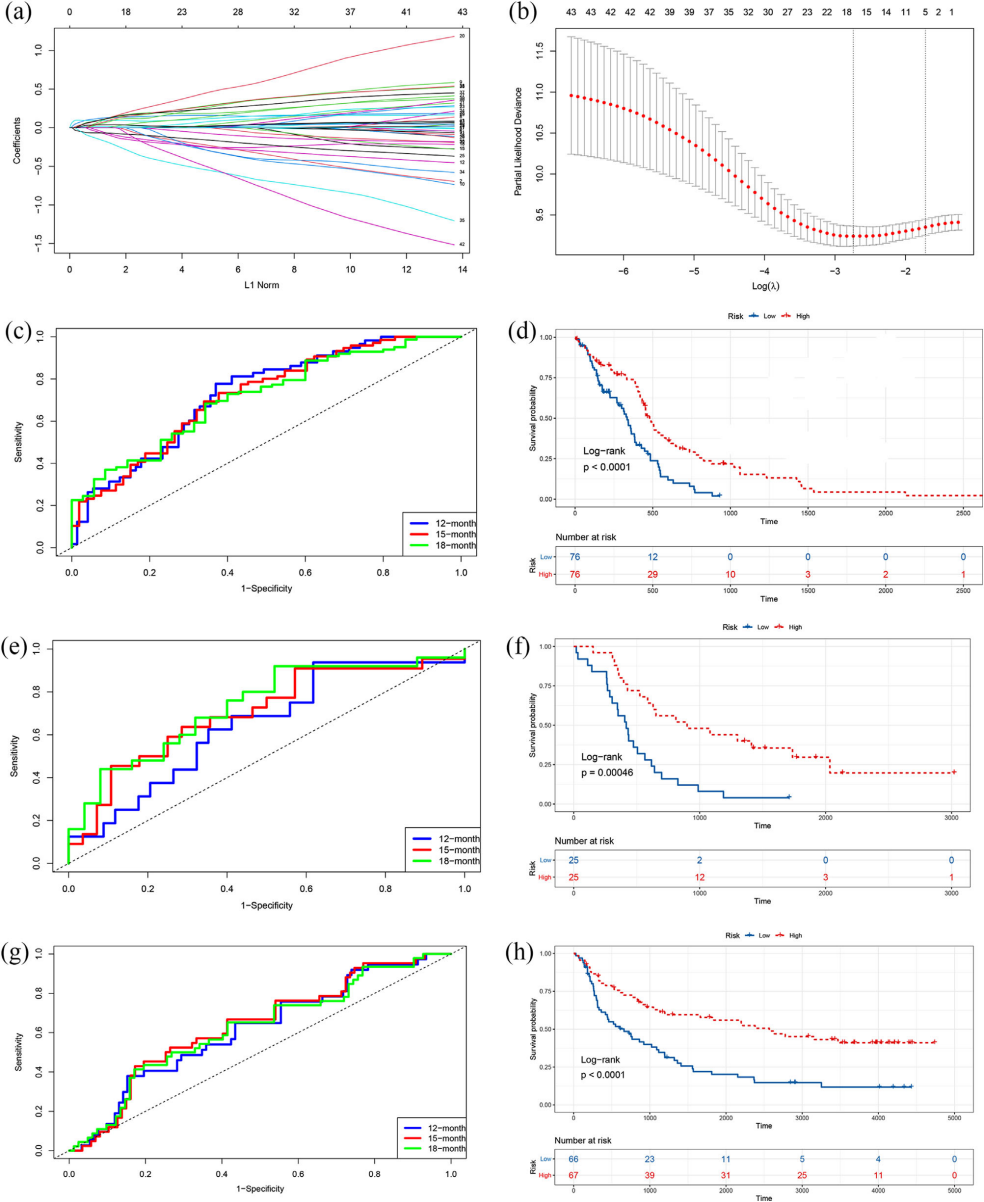
(a) least absolute shrinkage and selection operator (LASSO) coefficient profiles of the differentially expressed genes (DEGs) associated with the overall survival (OS) of glioblastoma (GBM) patients. (b) Partial likelihood deviance plotted versus log(lambda). The vertical dotted line indicates the lambda value with the minimum error and the largest lambda value where the deviance is within one SE of the minimum. (c) The receiver operating characteristic (ROC) curves for the risk score model in the training dataset. (d) The OS of patients in the five‐gene risk score model low‐ and high‐risk groups in the training dataset. (e) The ROC curves for the risk score model in the GSE43378. (f) The OS of patients in the five‐gene risk score model low‐ and high‐risk groups in the GSE43378. (g) The ROC curves for the risk score model in the mRNAseq_693. (h) The OS of patients in the five‐gene risk score model low‐ and high‐risk groups in the mRNAseq_693

After LASSO Cox penalized regression analysis in the training dataset (Figure 4a,b), we constructed a five‐gene (*LITAF*, *MTHFD2*, *NRXN3*, *OSMR*, and *RUFY2*)‐based risk score prognostic model. The risk score = 0.01301 × expression of *LITAF* – 0.03406 × expression of *MTHFD2* + 0.04864 × expression of *NRXN3* + 0.09675 × expression of *OSMR* – 0.00038 × expression of *RUFY2*. We performed time‐dependent ROC curve analysis to predict the 12‐, 15‐, and 18‐month survival, and the area under curves (AUCs) for 12‐, 15‐, and 18‐month OS were 0.728, 0.721, and 0.713, respectively (Figure 4c). In addition, the survival curve suggested that the high‐risk group showed a worse prognosis, compared to the low‐risk group, with *p* < .001 (Figure 4d).

### 3.6 Validation of the prognostic model

We used the dataset GSE43378 including 50 patients with complete OS information from GEO and the dataset mRNAseq_693 including 133 patients with complete OS information from CGGA as the external validation datasets to evaluate the robustness and effectiveness of our risk score prognostic model. We also performed time‐dependent ROC curve analysis to predict the 12‐, 15‐, and 18‐month survival. The AUCs for 12‐, 15‐, and 18‐month OS were 0.645, 0.701, and 0.733 in GSE43378, respectively (Figure 4e). The AUCs for 12‐, 15‐, and 18‐month OS were 0.616, 0.634, and 0.622 in mRNAseq_693, respectively (Figure 4g). Additionally, in Figure 4f,h, the survival curve indicated that the high‐risk group presented a worse prognosis than the low‐risk group (*p* < .001) in these two validation datasets. In summary, these results indicate the effective predictive capability of the risk score prognostic model constructed by integrated analysis of scRNA‐seq and bulk RNA‐seq datasets.

## 4 DISCUSSION

**FIGURE 5:**
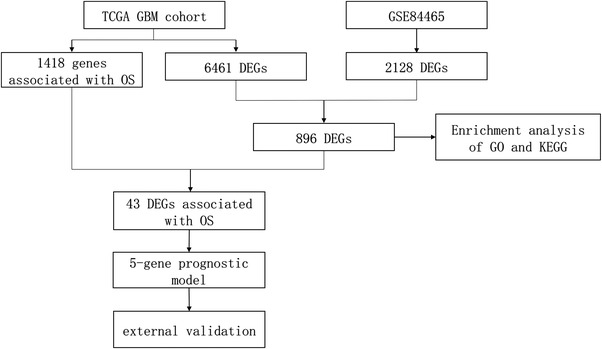
The process of constructing the five‐gene risk score model. First, 2128 differentially expressed genes (DEGs) and 6461 DEGs were identified from the GSE84465 and TCGA‐GBM datasets by differential expression analysis, respectively. In addition, using univariate Cox proportional hazards regression analysis, 1418 genes were identified to be associated with overall survival (OS) in glioblastoma (GBM) patients. Then, 43 DEGs associated with OS in GBM patients were obtained. Next, least absolute shrinkage and selection operator (LASSO) Cox penalized regression analysis was applied to construct a gene risk score model for prognosis prediction. Subsequently, the gene risk score model was constructed based on the five genes (*LITAF*, *MTHFD2*, *NRXN3*, *OSMR*, and *RUFY2*). Finally, the five‐gene risk score model was validated using validation datasets

Over years, there are increasing studies using public databases to predict survival in GBM. However, the DEGs were identified from bulk RNA‐seq in most studies, which failed to accurately reflect the difference between tumor cores and peripheral regions, thus weakening the predictive ability of the models (P. F. Chen et al., 2019; Xu et al., 2020; Zhang et al., 2020). On the other hand, analysis using the scRNA‐seq data could resolve gene expression at single‐cell resolution, allowing the classification and annotation of their expression in a cell‐type‐ or tissue‐specific manner. Neurons are regarded as the original cells of cancer cells in GBM (Friedmann‐Morvinski et al., 2012; Vescovi et al., 2006). In this study, we first obtained candidates DEGs from the scRNA‐seq dataset GSE84465, and then combined them with TCGA‐GBM to acquire the DEGs associated with OS. Finally, we constructed a risk score prognostic model and validated the model with the external dataset GSE43378. Figure 5 shows the flow of the study.

The results of KEGG analysis showed that DEGs were enriched in signaling pathways such as the MAPK pathway, the apelin pathway, and pathways involved in circadian entrainment. A report suggested that the MAPK signaling pathway plays an important role in GBM development and malignant progression through promoting GBM cell tumorigenicity (X. Chen et al., 2020). Another study also demonstrated that the apelin signaling pathway controls GBM angiogenesis and invasion (Mastrella et al., 2019). Our risk score model suggests that the five gene (*LITAF*, *MTHFD2*, *NRXN3*, *OSMR*, and *RUFY2*) might affect the OS of GBM patients through these pathways.

In the training dataset, we constructed a five‐gene (*LITAF*, *MTHFD2*, *NRXN3*, *OSMR*, and *RUFY2*)‐based risk score prognostic model. The risk score = 0.01301 × expression of *LITAF* – 0.03406 × expression of *MTHFD2* + 0.04864 × expression of *NRXN3* + 0.09675 × expression of *OSMR* – 0.00038 × expression of *RUFY2*. *LITAF* was identified as a transcription factor which activates the proinflammatory cytokine transcription in macrophages upon response to lipopolysaccharide. Recently, a study demonstrated that the *LITAF* expression is decreased in glioma tissues, which likely enhances the radiosensitivity of glioma cells through upregulating the FoxO1 pathway (Huang et al., 2019). *MTHFD2* is broadly required for cancer cell proliferation and viability as a metabolic enzyme and was overexpressed around the tumor regions with poor nutrient access in GBM patients, and the suppression of *MTHFD2* could cause cancer cell death (Tanaka et al., 2021). *NRXN3* belongs to a family of highly polymorphic neuronal‐specific cell surface proteins, and it was reported to promote glioma cell proliferation and migration under the regulation of Fox Q1 (Sun et al., 2013). Our results not only verified the relationship between *NRXN3* and GBM but also demonstrated the effectiveness of using *NRXN3* as an important indicator for prognosis prediction in GBM patients. *OSMR* is a member of the interleukin‐6 receptor family, and a previous study reported that it regulates GBM tumor growth through orchestrating a feed‐forward signaling mechanism with EGFRvIII and *STAT3* to promote tumorigenesis (Jahani‐Asl et al., 2016). Furthermore, another study showed that *OSMR* conferred resistance to ionizing radiation via regulation of oxidative phosphorylation and that loss of *OSMR* sensitized GBM tumors to ionizing radiation therapy (Sharanek et al., 2020). However, the function of *RUFY2* remains unknown.

In the validation dataset GSE43378, the AUCs for 12‐, 15‐, and 18‐month OS were 0.645, 0.701, and 0.733, respectively. MRNAseq_693 downloaded from CGGA, whose all GBM samples were Chinese patients, was selected as the other validation dataset. The AUCs for 12‐, 15‐, and 18‐month OS were 0.616, 0.634, and 0.622 in mRNAseq_693, respectively. In addition, the survival curve suggested that the high‐risk group exhibited a worse prognosis than the low‐risk group (*p* < .001). These results indicate the predictive capability of the risk score prognostic model. Unlike the previous prognostic signature, our risk score prognostic model was constructed by integrating the scRNA‐seq dataset and bulk RNA‐seq dataset. This finding could reflect the effect of these genes on GBM patient prognosis. Based on the risk score, the survival probabilities of an individual can be queried based on the level of the five genes (*LITAF*, *MTHFD2*, *NRXN3*, *OSMR*, and *RUFY2*). For patients with high risk of progression to severe conditions, it is important for them to receive adequate attention and care during treatments; therefore, our model will be a valuable tool to provide a good reference for clinicians.

In summary, we constructed a novel prognostic model to predict the survival in GBM patients though integrative analysis of a scRNA‐seq dataset and a bulk RNA‐seq dataset. Our findings have the potential to advance the development of new therapeutics for the treatment of GBM and improve the clinical outcomes for GBM patients. However, there are some limitations in our study. First, clinical characteristics were not taken into account in our model. In the future, we can improve our model by integrating clinical characteristics from the analysis of patients with more comprehensive clinical information. In addition, our model was constructed and validated using public databases, and it would also be helpful to validate the model with private clinical or experimental datasets in the further.

Figure S1: GO functional and KEGG pathway analysis. (a) Summary of the differentially expressed genes and GO pathway enrichment. Red, blue, and green bars represent the biological process, cellular component, and molecular function categories, respectively. The height of the bar represents the number of differentially expressed genes observed in each category. (b) The top 10 pathways involving the differentially expressed genes.

## CONFLICT OF INTEREST

The authors declare no conflict of interest.

## AUTHOR CONTRIBUTIONS

*Methodology, software, validation, data curation, and writing‐original draft*: Wenwen Lai. *Methodology, formal analysis, and visualization*: Defu Li. *Software and Data curation*: Jie Kuang. *Supervision, methodology, and formal analysis*: Libin Deng. *Conceptualization, project administration, and funding acquisition*: Quqin Lu.

### PEER REVIEW

The peer review history for this article is available at https://publons.com/publon/10.1002/brb3.2575.

## Supporting information

Supporting Information

## Data Availability

Data were downloaded from the TCGA, GEO, and CGGA website.
