# Supplementary figures and images for: Integrated analysis of single‐cell RNA‐seq dataset and bulk RNA‐seq dataset constructs a prognostic model for predicting survival in human glioblastoma
Source: Brain Behav. 2022 Apr 16;12(5):e2575. doi: 10.1002/brb3.2575 (PMC9120724; doi:10.1002/brb3.2575)

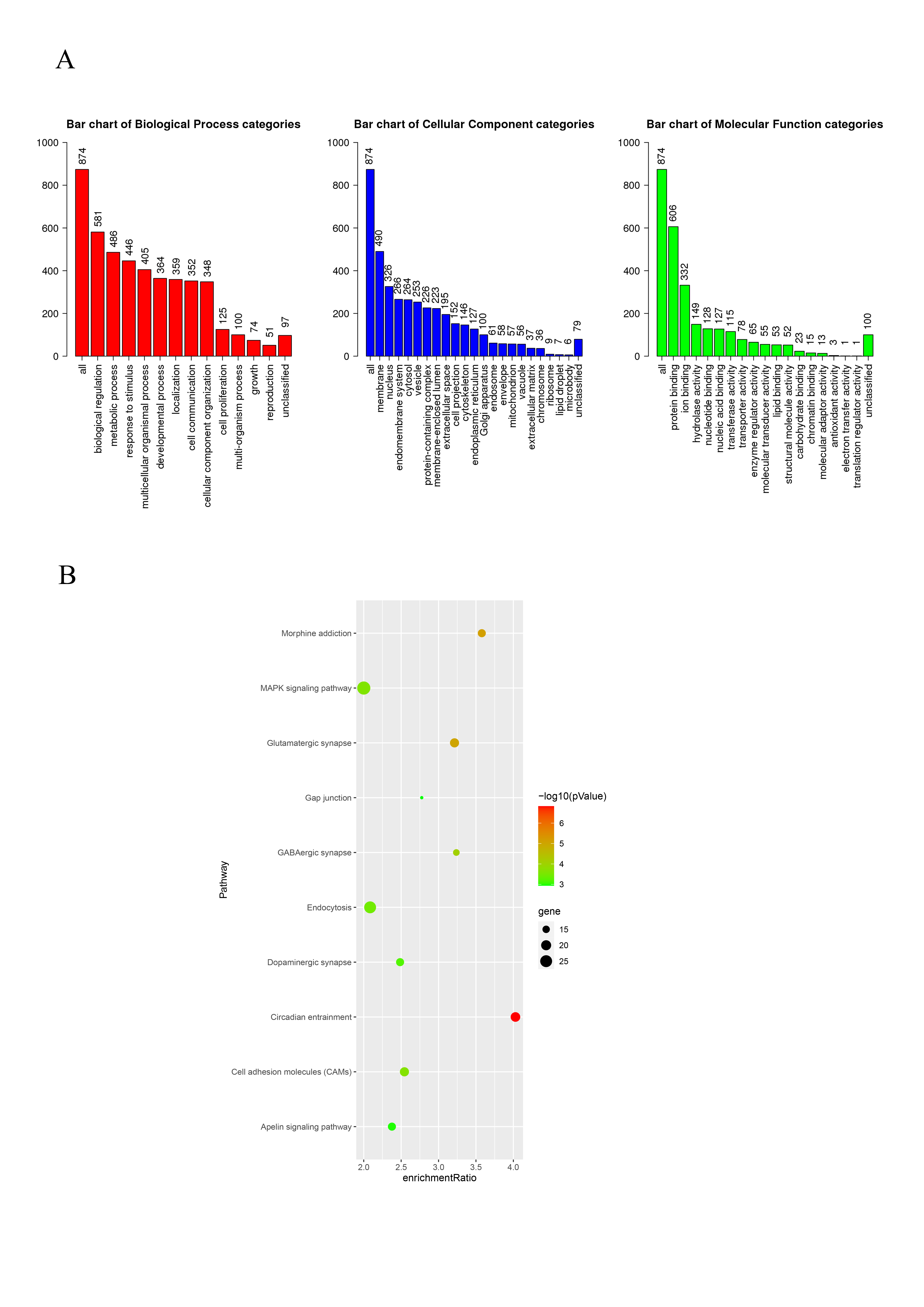

Supplement: Supplementary file 1 — Supporting Information [file BRB3-12-e2575-s001.tif]
